# Dual targeting PET tracer [^68^Ga]Ga-FAPI-RGD in patients with lung neoplasms: a pilot exploratory study

**DOI:** 10.7150/thno.86007

**Published:** 2023-05-15

**Authors:** Rongxi Wang, Vivianne Jakobsson, Jiarou Wang, Tianzhi Zhao, Xingtong Peng, Bowen Li, Jianchao Xue, Naixin Liang, Zhaohui Zhu, Xiaoyuan Chen, Jingjing Zhang

**Affiliations:** 1 Department of Nuclear Medicine, State Key Laboratory of Complex Severe and Rare Diseases, Beijing Key Laboratory of Molecular Targeted Diagnosis and Therapy in Nuclear Medicine, Peking Union Medical College Hospital, Chinese Academy of Medical Sciences, Peking Union Medical College, Beijing, China.; 2 Departments of Diagnostic Radiology, Yong Loo Lin School of Medicine, National University of Singapore, Singapore, 119074, Singapore.; 3 Clinical Imaging Research Centre, Centre for Translational Medicine, Yong Loo Lin School of Medicine, National University of Singapore, Singapore 117599, Singapore.; 4 Nanomedicine Translational Research Program, NUS Center for Nanomedicine, Yong Loo Lin School of Medicine, National University of Singapore, Singapore 117597, Singapore.; 5 Eight-Year Program of Clinical Medicine, Peking Union Medical College Hospital (PUMCH), Chinese Academy of Medical Sciences (CAMS) and Peking Union Medical College (PUMC), Beijing 100730, China.; 6 Department of Thoracic Surgery, Peking Union Medical College Hospital, Chinese Academy of Medical Sciences and Peking Union Medical College, Beijing 100730, China.; 7 Chinese Academy of Medical Sciences and Peking Union Medical College, Beijing 100730, China.; 8 Departments of Chemical and Biomolecular Engineering and Biomedical Engineering, Faculty of Engineering, National University of Singapore, Singapore 117597, Singapore.

**Keywords:** [^68^Ga]Ga-FAPI-RGD, [^18^F]FDG, Lung neoplasm, FAP, Integrin α_v_β_3_

## Abstract

**Rationale:** Early discovery, accurate diagnosis, and staging of lung cancer is essential for patients to receive appropriate treatment. PET/CT has become increasingly recognized as a valuable imaging modality for these patients, but there remains room for improvement in PET tracers. We aimed to evaluate the feasibility of using [^68^Ga]Ga-FAPI-RGD, a dual-targeting heterodimeric PET tracer that recognizes both fibroblast activation protein (FAP) and integrin α_v_β_3_ for detecting lung neoplasms, by comparing it with [^18^F]FDG and single-targeting tracers [^68^Ga]Ga-RGD and [^68^Ga]Ga-FAPI.

**Methods:** This was a pilot exploratory study of patients with suspected lung malignancies. All 51 participants underwent [^68^Ga]Ga-FAPI-RGD PET/CT, of which: 9 participants received dynamic scans, 44 participants also underwent [^18^F]FDG PET/CT scan within two weeks, 9 participants underwent [^68^Ga]Ga-FAPI PET/CT scan and 10 participants underwent [^68^Ga]Ga-RGD PET/CT scan. The final diagnosis was made based on histopathological analyses and clinical follow-up reports.

**Results:** Among those who underwent dynamic scans, the uptake of pulmonary lesions increased over time. The optimal timepoint for a PET/CT scan was identified to be 2 h post-injection. [^68^Ga]Ga-FAPI-RGD had a higher detection rate of primary lesions than [^18^F]FDG (91.4% *vs.* 77.1%, *p* < 0.05), higher tumor uptake (SUVmax, 6.9 ± 5.3 *vs.* 5.3 ± 5.4, *p* < 0.001) and higher tumor-to-background ratio (10.0 ± 8.4 *vs.* 9.0 ± 9.1, *p* < 0.05), demonstrated better accuracy in mediastinal lymph node evaluation (99.7% *vs.* 90.9%, *p* < 0.001), and identified more metastases (254 *vs.* 220). There was also a significant difference between the uptake of [^68^Ga]Ga-FAPI-RGD and [^68^Ga]Ga-RGD of primary lesions (SUVmax, 5.8 ± 4.4 *vs.* 2.3 ± 1.3, *p* < 0.001).

**Conclusion:** In our small scale cohort study, [^68^Ga]Ga-FAPI-RGD PET/CT gave a higher primary tumor detection rate, higher tracer uptake, and improved detection of metastases compared with [^18^F]FDG PET/CT, and [^68^Ga]Ga-FAPI-RGD also had advantages over [^68^Ga]Ga-RGD and was non-inferior to [^68^Ga]Ga-FAPI. We thus provide proof-of-concept for using [^68^Ga]Ga-FAPI-RGD PET/CT for diagnosing lung cancer. With the stated advantages, the dual-targeting FAPI-RGD should also be explored for therapeutic use in future studies.

## Introduction

Lung cancer is a significant problem globally, with the highest morbidity and mortality of all cancers 1. Early discovery, accurate diagnosis, and staging are essential for patients to receive appropriate treatment and significantly affect prognosis. The standard-of-care screening and imaging of lung cancer is done with conventional X-rays and low-dose chest CT scans. However, X-ray scans are inferior in many aspects, and low-dose chest CT evaluates the morphology of lesions only; clinicians are thus relying more and more on molecular imaging modalities such as PET/CT scan, which integrates functional and anatomical imaging 1, 2.

^18^F-Fluorodeoxyglucose ([^18^F]FDG) remains the most widely used PET tracer for the diagnosis and staging of malignancies, including lung cancer 3-5. However, there are well-documented limitations of [^18^F]FDG, such as false-negative uptake in the primary neoplasms inside less invasive subtypes and/or early stages of lung cancer and false-positive uptakes in other acute or chronic infectious diseases 6-11. Additionally, [^18^F]FDG has the significant drawback of low sensitivity for brain metastases due to its high uptake in healthy brain tissue, giving low contrast, and insensitivity for bone metastases, due to low FDG uptake in osteogenic bone metastases 12, 13. Thus, a more accurate and reliable diagnosis modality at an early stage is crucial and inspires the development of alternative novel PET tracers for patients with lung cancer.

Fibroblast activating protein (FAP) is a prolyl endopeptidase and a type II transmembrane glycoprotein 14 reported to be overexpressed in over 90% of epithelial tumors, including human lung tumors 15. Solid tumors primarily consist of tumor microenvironment components such as FAP expressing cancer-associated fibroblasts (CAFs) and extracellular fibrotic tissue 16. A growing number of FAP targeting tracers have been developed for imaging and therapeutic purposes, the most common being quinoline-based FAP inhibitors (FAPIs) 17-20. FAPIs have so far shown promising potential as PET tracers, with FAPI-based PET/CT demonstrating a greatly improved tumor uptake, sensitivity, tumor-to-background (T/B) ratio, and overall tumor detection rate compared to [^18^F]FDG PET/CT in various cancer types 21.

Integrin α_v_β_3_ is highly expressed on activated endothelial cells, newborn blood vessels, and several types of tumor cells 22, 23. Integrin α_v_β_3_ binding radiotracers based on Arg-Gly-Asp (RGD) sequence have been developed for lesion and neovascularization detection with proven benefits to the diagnosis, staging, and therapeutic efficacy evaluation in participants with, for example, breast cancer, glioma, and lung cancer 23.

Single-target receptors, such as monomer FAPI and RGD radiotracers, still have some limitations for clinical application. The complex heterogeneity of the tumor for different cancer types leads to deficient uptake of a single-targeting tracer. Meanwhile, the expression level of a particular receptor may change as the tumor grows or differentiates. In addition, due to the small molecular size, the single-targeting tracer exhibits fast clearance, potentially leading to missing lesions. In attempts to overcome the current limitations of single-target tracers, new designs, including multimeric approaches like heterodimers with binding affinity to multiple targets on a single entity, have been developed 24-26. Based on previous studies targeting either FAP or integrin α_v_β_3_ for lung cancer imaging with reported advantages 27-30, we synthesized a dual-targeting dimer, [^68^Ga]Ga-FAPI-RGD. Our published preliminary study showed promises *in vitro* and *in vivo*. A pilot investigation has been conducted in 6 cancer participants, where [^68^Ga]Ga-FAPI-RGD showed a notably higher tumor uptake in different cancer types at all times than either the monomeric FAPI or RGD tracers separately 26, 31. We hypothesize that this dual-targeting tracer will again, in this larger cohort, outperform [^18^F]FDG and the single-target tracers [^68^Ga]Ga-RGD and [^68^Ga]Ga-FAPI with improved sensitivity and specificity for detecting lung cancer lesions. Therefore, we aim to explore further [^68^Ga]Ga-FAPI-RGD as a new PET tracer, evaluating its uptake in the primary and metastatic lung neoplasms and comparing the detection efficiency and diagnosis of lung cancer with [^18^F]FDG and the single-target tracers, [^68^Ga]Ga-RGD and [^68^Ga]Ga-FAPI.

## Materials and Methods

### Participants

This pilot exploratory study was approved by the clinical research ethics committee of Peking Union Medical Hospital (I-22PJ249) and conducted in accordance with the 1964 Declaration of Helsinki and its later amendments or comparable ethical standards. The study was registered at ClinicalTrials.gov (National Institutes of Health) (NCT05543954). All participants provided written informed consent.

Participants were recruited from September 2022 to November 2022 at Peking Union Medical Hospital. Inclusion criteria for participants were as follows: 1) aged 18 years and above); 2) no gender preference; 3) all patients were referred by the thoracic surgeon with suspected lung neoplasms on chest CT scan as part of routine screening or with symptoms (only participants who had no other prior treatment within 3 months were eligible for enrollment in receiving both [^68^Ga]Ga-FAPI-RGD PET/CT and [^18^F]FDG PET/CT; 4) biopsy or surgical specimens available for histopathological confirmation of lung neoplasms (participants with previous 3-month follow-up images showing an increase in lesion size were also included); 5) participants had to be able to understand and sign an informed consent form; 6) participants had to be available for the required follow-up time and have more than 6 months of predicted survival. Exclusion criteria included: 1) patients with a 2^nd^ primary tumor; 2) pregnancy or breastfeeding; 3) severe liver or kidney diseases, serum creatine > 3.0 mg/dl, liver/hepatic enzymes of ≥ 5 times higher than normal upper limit/maximum (ULN upper limit of normal); 4) claustrophobia or other contraindications for PET/CT scans.

Multiple imaging modalities, *e.g.*, MRI of the brain, chest CT, whole body bone scan, and hybrid imaging PET/CT, served as supporting evidence for diagnosing lymph node metastases and other distant metastases. Participant information was collected, including a family history of cancer, smoking history, and previous treatment history (surgery, systemic therapies, and radiation therapy). We compared [^68^Ga]Ga-FAPI-RGD PET/CT and [^18^F]FDG PET/CT for the number of lesions identified, lesion locations, and lesion sizes. The final diagnosis was histopathologically confirmed with a biopsy or surgical specimen as the gold standard reference. Only a small portion of suspected lesions was analyzed, considering that some cancer participants were in the final stage of their disease. Imaging follow-ups were conducted if the histopathological analysis was unavailable. The participant data is recorded in **Table 1**.

### Radiochemistry

The structure of this dual-targeting new tracer has been described in detail in our previous publication 26. All FAPI-RGD precursors were provided in-kind by Yantai Lannacheng Biotechnology (Yantai, China). The FAPI-04 (DOTA-FAPI-04) and RGD (NOTA-3PRGD2) precursors were purchased from CSBio (Shanghai, China) for research and development purposes. Radiolabeling followed a standard procedure reported in the preclinical studies. Radiochemical purity was confirmed to be greater than 95% with thin-layer chromatography (TLC). The final product was diluted with saline to optimal concentration and confirmed sterile before injection into participants. The preparation of [^18^F]FDG followed standard clinical practice.

### [^68^Ga]Ga-FAPI-RGD and [^18^F]FDG PET/CT Imaging

All participants were intravenously injected with [^68^Ga]Ga-FAPI-RGD (approximately 1.5-2 MBq/kg body weight) without special preparation. Dynamic acquisition was performed for 9 participants at different time points (5-15 min, 20-30 min, 35-45 min, 50-60 min, 70-90 min, 100-120 min, 130-150 min, 160-180 min) after injection while the others underwent PET/CT scan 2 h p.i. only.

The participants undergoing [^18^F]FDG PET/CT were instructed to fast for a minimum of 5 h before the scan since a normal blood glucose level was required and then intravenously injected with 5.55 MBq/kg [^18^F]FDG. Images were acquired with a dedicated PET/CT scanner (PoleStar m660, Sinounion Healthcare Inc., Beijing, China). Participants underwent scans in supine positions. For dynamic scanning, a low-dose CT scan (120 keV, 120 mA) was performed from the skull base to the feet, and the other scans were conducted from the skull base to the proximal thigh for attenuation correction as well as anatomical localization. PET scans were done under 3D acquisition mode with several bed positions (1-2 min/bed position for dynamic scans, 2 min/bed position for other participants; 192 x 192 matrix). The acquired imaging data were processed with a 3D ordered subset expectation maximization (OSEM) and time of flight (TOF) reconstruction algorithm.

### [^68^Ga]Ga-FAPI and [^68^Ga]Ga-RGD PET imaging

[^68^Ga]Ga-RGD PET/CT was performed on 10 participants, and [^68^Ga]Ga-FAPI PET/CT on 9 participants for comparison with [^68^Ga]Ga-FAPI-RGD PET/CT. [^68^Ga]Ga-RGD and[^68^Ga]Ga-FAPI PET/CT was performed 1 h after administration of radiopharmaceuticals (1.5-2 MBq/kg) following the same operation procedure and imaging analysis as [^68^Ga]Ga-FAPI-RGD PET/CT.

### Image analysis

Image analysis was performed with MIM software (Version 7.1.4, Cleveland, OH, USA) to interpret the coronal, transverse, and sagittal planes. PET images were visually analyzed independently by two experienced nuclear medicine physicians (Z.Z. and J.Z. with more than 10 years of expertise in nuclear oncology; they were different from the physicians who previously recruited the patients and interpreted the images) on a frame-to-frame basis, blind to the participant's medical history, other examinations, and clinical information. CT-suspected lesions were diagnosed as benign if no pathological uptake of any tracer was observed. VOI (volume of interest), SUVmax (standardized maximal uptake value), and SUVmean of each lesion were measured for semi-quantitative analysis of the PET scan images. For background comparison (tumor-to-background ratio), SUV mean and SUVmax were measured for the brain, thyroid, submandibular gland, lung parenchyma, mediastinal blood pool, liver, spleen, pancreas, kidneys, and red bone marrow. Primary tumor calculations were based on 40% of SUVmax at lesion regions in the PET images. SUVmean of control areas were used as background values, *e.g.*, contralateral lung with no lesions for lung neoplasms and aortic arch area for lymph nodes. For [^68^Ga]Ga-PET and [^18^F]FDG PET/CT imaging, lymph node metastasis was defined as a non-physiological uptake higher than the neighboring tissue. The participants who were unavailable for follow-ups were not included in this image analysis.

### Dosimetry estimation

The dosimetry estimation was calculated with hybrid dosimetry software from Hermes Medical Solutions, Sweden. Major organs were manually drawn for each time point, and bioaccumulation data were obtained. Olinda/EXM Version 2.2.0 was used for fitting the time-activity curves and dosimetry calculations. Source organs included kidneys, liver, spleen, urinary bladder content, lumbar vertebrae (L2-L4) for red bone marrow, myocardium, lungs, brain, pancreas, thyroid gland, salivary gland, and stomach content. Lumbar vertebrae (L2-L4) were considered to make up 6.7% of bone marrow and were used to determine the red marrow absorption with 3D volumetric analysis. We calculated the time-activity curve based on the regions of interest (ROIs) of the above organs drawn on a selected imaging time point and copied to other time points. The curve was fitted by mono- or bi-exponential curve fitting parameters, and the residence time in the source organs was then derived. With this input in the built-in adult human model, the absorbed dose of all organs and total body effective dose were generated (mSv/MBq). A Time-activity curve was then plotted with GraphPad Prism 9.0.

### IHC staining

The pathology sections of lung neoplasms and some lymph nodes were collected from participants' surgical resection specimens at Peking Union Medical College Hospital. For histopathological analysis, the paraffin-embedded specimens were stained with a FAP antibody (anti-rabbit, diluted to 1:250; Abcam, ab207178) and an integrin β_3_ antibody (anti-rabbit, diluted to 1:100; CST, #13166). Representative samples were selected based on the quality and quantity of the tissue. To semi-quantify the expression of FAP and integrin β_3_, a score was given to a randomly picked section containing 5 malignant cell clusters (40X complete high magnification field) based on the staining intensity and percentage area by an experienced pathologist. The analysis followed previous reports 32, 33, with adjacent normal tissue stained negative for FAP and integrin β_3_ used as a negative control.

### Statistical analysis

All statistical analyses were based on Prism 9.0 (GraphPad, San Diego, CA) and SPSS 22.0 software from IBM Corp., Armonk, NY, USA. All continuous data were expressed as mean ± standard deviation. Non-continuous data were presented as counts or percentages. The Mc-Nemar test was used to evaluate the difference in detection rates of primary lung neoplasms between [^68^Ga]Ga-FAPI-RGD and [^18^F]FDG PET/CT. Comparison of the tumor-to-background ratio of the number of SUVs and metastatic lesions detected by [^68^Ga]Ga-FAPI-RGD and [^18^F]FDG, [^68^Ga]Ga-FAPI-RGD and two single-target imaging agents used Wilcoxon signed-rank test. *P*-value < 0.05 was considered statistically significant. The Spearman correlation coefficient was used to measure the correlation between the SUVs and the IHC staining score.

## Results

### Participants characteristics

**Figure 1:**
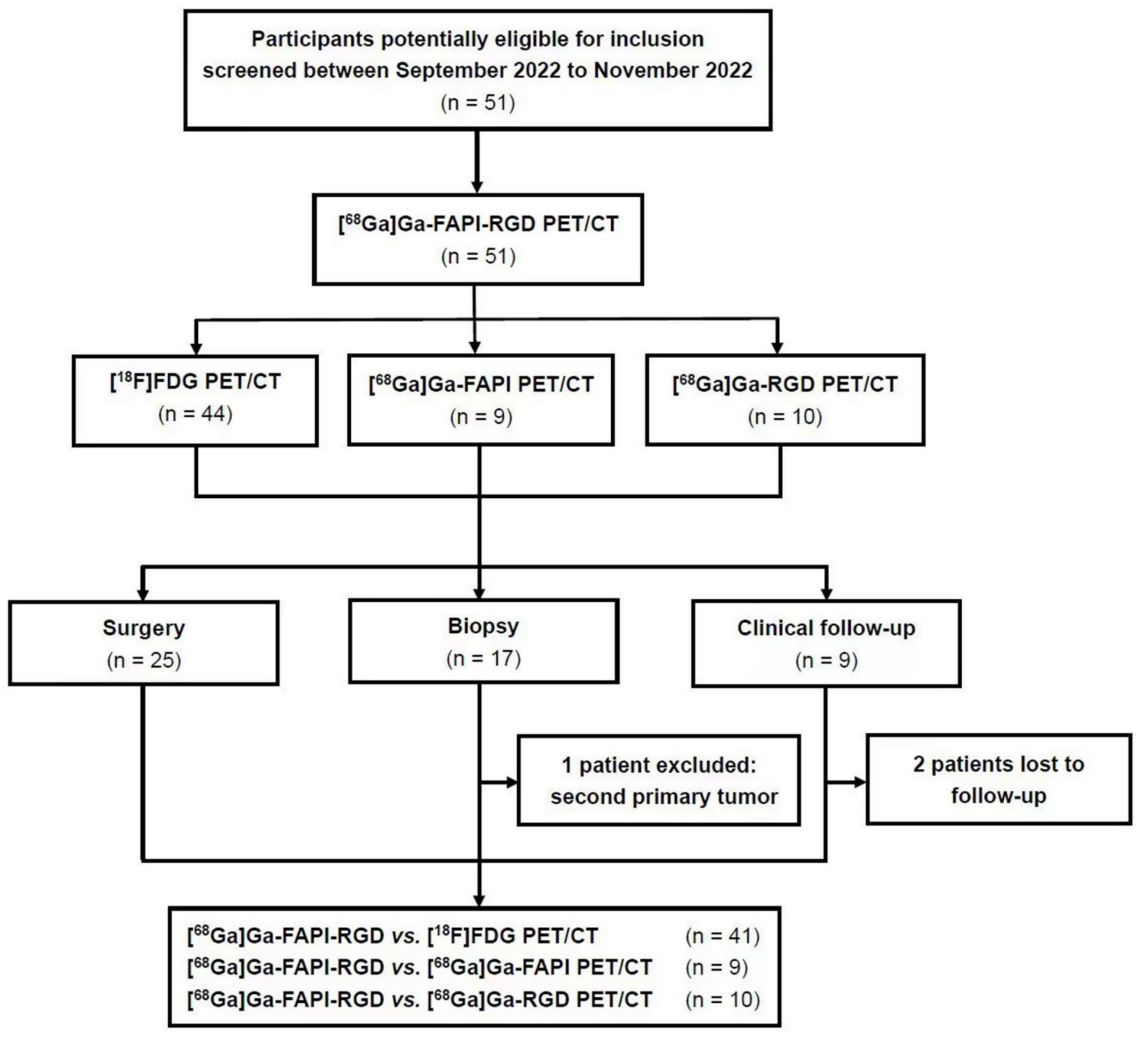
Flow diagram showing participant selection details. FAPI = fibroblast activation protein inhibitor, RGD = Arg-Gly-Asp, FDG = fluorodeoxyglucose, ^68^Ga = gallium 68, ^18^F = fluorine 18.

Fifty-one participants with suspected lung cancer (21 male participants, 30 female participants; range, 30 - 79 years; average age, 61 ± 13 years, median age, 66 years) were enrolled in this study and underwent [^68^Ga]Ga-FAPI-RGD PET/CT scan with the injected activity of 3.2 ± 0.4 mCi (118.8 ± 14.8 MBq) **(Figure 1)**. The administration was well tolerated, and no adverse events were observed or reported in any participant during the radiopharmaceutical administration. 19 participants had a smoking history (17 males and 2 females). Within two weeks, 44 participants underwent [^18^F]FDG PET/CT scans, 9 participants underwent [^68^Ga]Ga-FAPI PET/CT scans and 10 participants underwent [^68^Ga]Ga-RGD PET/CT scans for head-to-head comparisons. A total of 50 biopsy specimens from 42 participants were acquired through CT-guided transthoracic needle biopsy or surgery. After histopathological analysis, 40 lesions were confirmed to be malignant neoplasms, 4 were benign tumors (2 atypical adenomatous hyperplasia, 1 pulmonary hamartoma, and 1 pulmonary leiomyoma), 5 were inflammatory lesions, and 1 was the second primary tumor. 8 primary pulmonary lesions were also identified as malignant by their [^18^F]FDG or [^68^Ga]Ga-FAPI-RGD uptake, confirmed with an increase in lesion size during the 3-month CT imaging follow-up. Two patients were unavailable to be followed up due to loss of contact. Participants' information is summarized in **Table 1.**

### 3-hour dynamic acquisition of [^68^Ga]Ga-FAPI-RGD PET/CT

**Figure 2:**
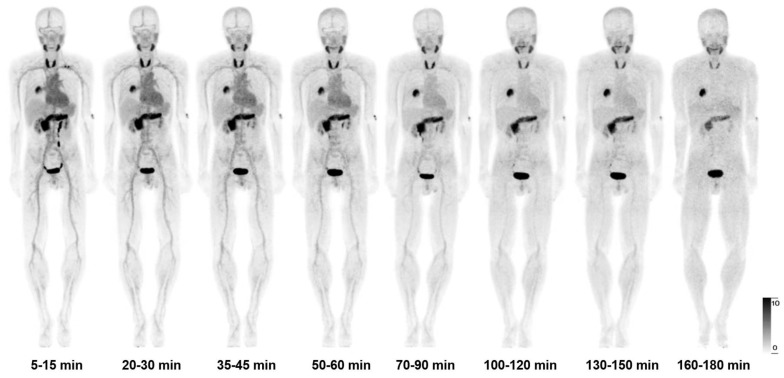
The maximal intensity projection (MIP) images of [^68^Ga]Ga-FAPI-RGD PET/CT over time of participant No. 1.

**Figure 3:**
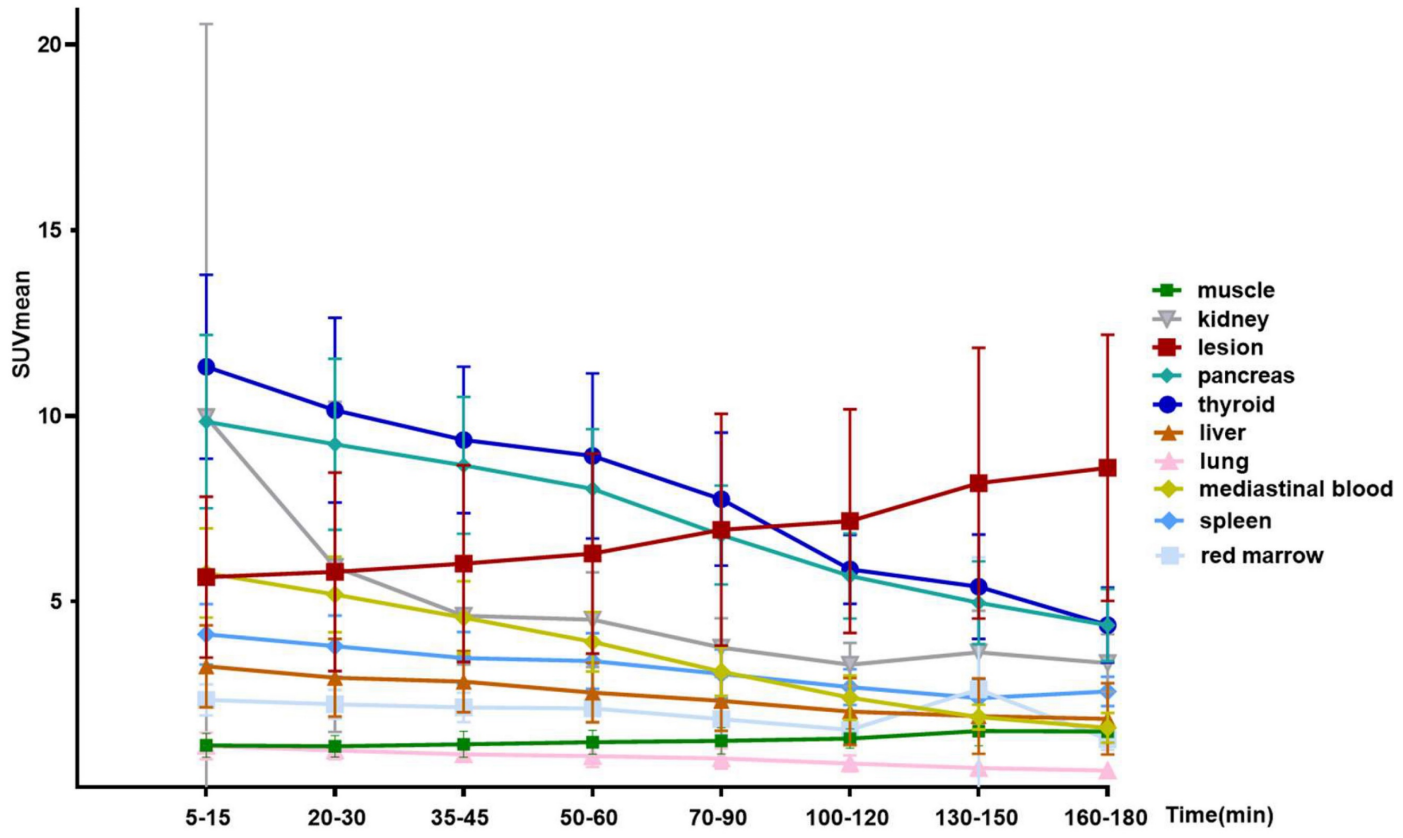
The average 3-h time-activity curves (TACs) from 9 participants of [^68^Ga]Ga-FAPI-RGD in normal organs and tumors over time.

After intravenous administration, [^68^Ga]Ga-FAPI-RGD was rapidly cleared *via* kidneys. There was high accumulation in the thyroid gland, pancreas, and urinary bladder. At 60 min p.i, the SUVmean values for the thyroid gland and pancreas were 8.9 ± 2.2 and 8.0 ± 1.6, respectively. At 120 min p.i., the SUVmean for the thyroid gland and pancreas were 5.9 ± 0.9 and 5.7 ± 1.1. Medium uptake was observed in the mediastinal blood pool (SUVmean 2.4), liver (SUVmean 2.0), and spleen (SUVmean 2.7). Brain and muscle had relatively low background activity (**Table S1**), in accordance with previous reports. **Figure 2** shows the maximal intensity projection (MIP) images of [^68^Ga]Ga-FAPI-RGD PET/CT over time of participant No. 1. **Figure 3** shows the average 3-h time-activity curves (TACs) from 9 participants of [^68^Ga]Ga-FAPI-RGD in normal organs and tumor over time. The lung tumor lesions could be clearly observed at the first time point. The SUVmax and SUVmean of normal organs decreased over time, and for tumor lesions, both increased. SUVmean values for tumors were higher than that of the neighboring tissue at around 90 min p.i., which made the tumor more clearly visible and continued to increase over 3 hours. Considering that multiple metastatic lesions throughout the body may bias the overall dose estimation for major organs, we thus excluded two patients with multiple metastases and 7 participants to calculate the estimated organ-absorbed doses of [^68^Ga]Ga-FAPI-RGD (**Table 2**). The urinary bladder wall had the highest absorbed dose, 8.00E-02 ± 3.3E-02 mSv/MBq, due to its function as an excretory organ where the radioactive substance accumulated following renal filtration, followed by the thyroid gland (7.8E-02 ± 5.1E-02 mSv/MBq) and pancreas (7.3E-02 ± 2.5E-02 mSv/MBq). The total body absorbed dose and the effective dose were calculated to be 1.22E-02 ± 1.1E-03 mSv/MBq and 2.02E-02 ± 1.80E-03 mSv/MBq, respectively.

### Comparison of [^18^F]FDG and [^68^Ga]Ga-FAPI-RGD in primary lung neoplasms

**Figure 4:**
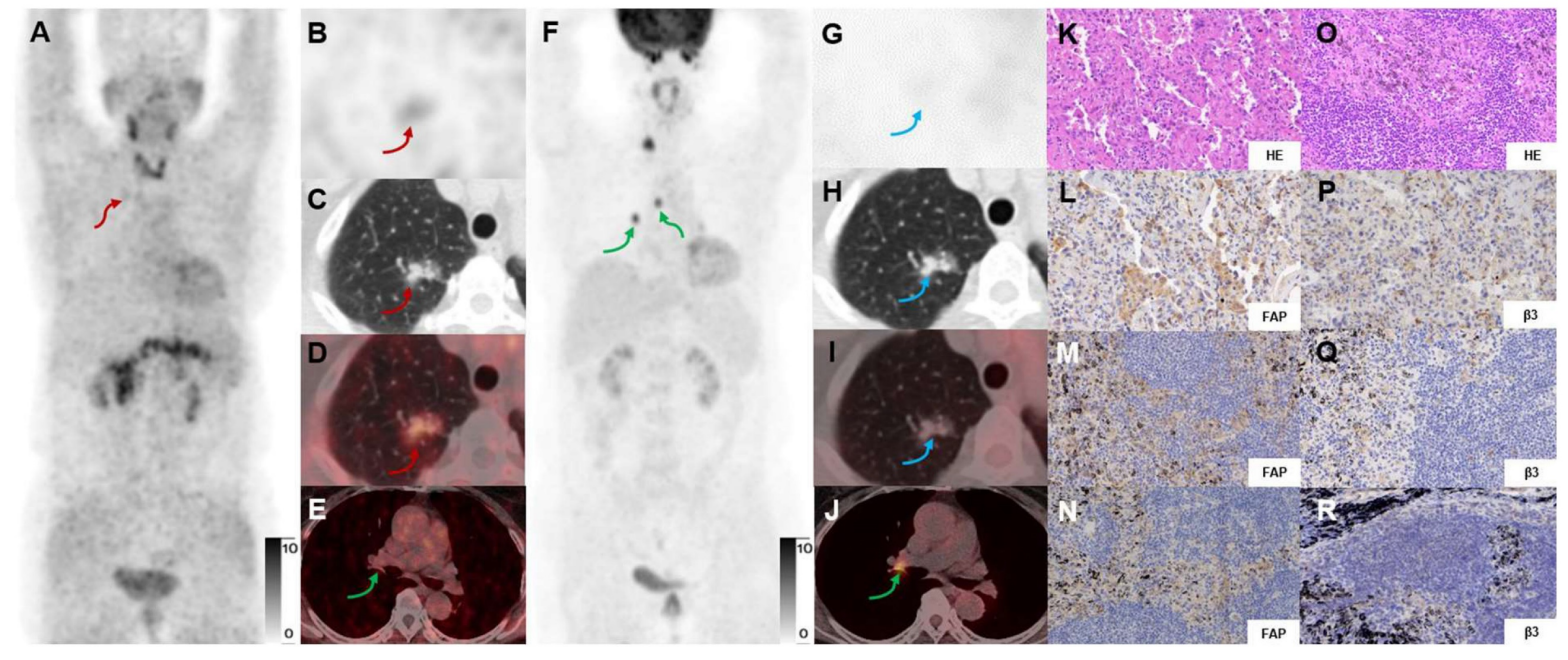
In a 69-y-old woman with IAC (Participant No.11), [^68^Ga]Ga-FAPI-RGD PET/CT (A-E) showed moderate uptake in the right upper lung lesion (red arrows). In contrast, [^18^F]FDG PET/CT (F-J) showed low uptake (blue arrows) (SUVmax, 4.0 *vs.* 1.0). HE and immunohistochemical staining showed low expression of FAP and β_3_ (K, L, P). However, the high uptake in lymph nodes (green arrows) shown on [^18^F]FDG but not on [^68^Ga]Ga-FAPI-RGD PET/CT were confirmed as inflammatory lymph nodes by postoperative pathology (O). Immunohistochemical staining showed low expression of FAP and β_3_ (M, N, Q, R). [^18^F]FDG-PET scan showed a high uptake lesion in the right lower neck on the right side of the thyroid gland.

**Figure 5:**
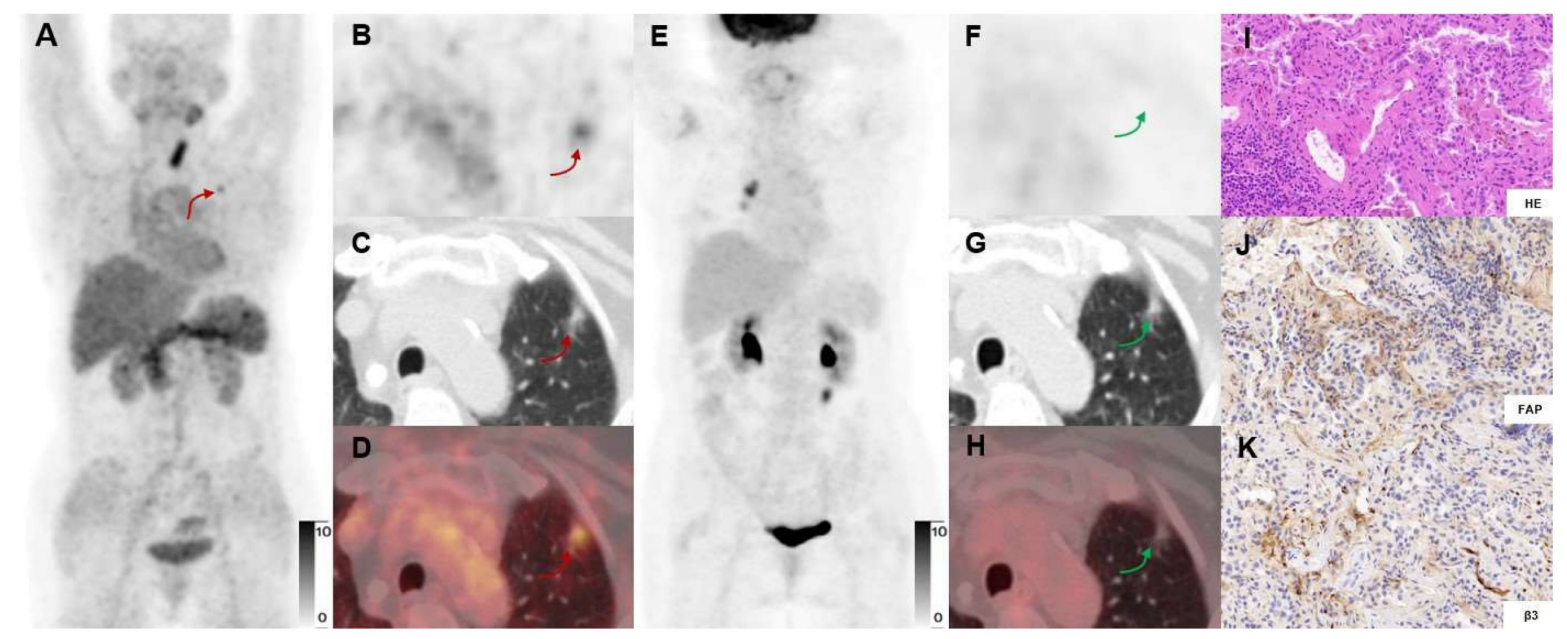
In a 71-y-old woman with MIA (Participant No.13) who previously had undergone a right thyroid resection, [^68^Ga]Ga-FAPI-RGD PET/CT (A-D) showed high uptake in the left upper lung lesion (red arrows), while [^18^F]FDG PET/CT (E-H) showed no uptake (green arrows). Immunohistochemical staining showed moderate expression of FAP and β_3_ by postoperative pathology (J, K). The right mediastinal lymph node with [^18^F]FDG uptake was considered an inflammatory lymph node.

There was a significant difference in the detection rate of malignant neoplasms between [^18^F]FDG and [^68^Ga]Ga-FAPI-RGD (**Figures 4-5**). Among 32 primary lesions in 24 surgery participants, [^68^Ga]Ga-FAPI-RGD had a higher detection rate (21 out of 24 malignant neoplasms) than [^18^F]FDG (16 out of 24). In the 8 cases of either inflammatory or benign neoplasms, 5 had an uptake of both tracers, and 3 had neither, both with a false-positive rate of 62.5%.

28 positive lymph nodes were identified in 6 participants by [^18^F]FDG, while [^68^Ga]Ga-FAPI-RGD only identified 1 positive lymph node in 1 participant. Surgery was performed on 24 participants, and 307 hilar and mediastinal lymph nodes were surgically removed and confirmed to be all benign inflammatory lymph nodes by postoperative pathological evaluation. It could thus be deduced that [^68^Ga]Ga-FAPI-RGD had significantly higher specificity than [^18^F]FDG in the detection of mediastinal lymph nodes (23/24 for [^68^Ga]Ga-FAPI-RGD *vs.* 18/24 for [^18^F]FDG, *p* < 0.001 in the number of patients, and 306/307 *vs.* 279/307, *p* < 0.001, respectively in the number of lymph nodes).

Of a total of 44 lesions with pathological confirmation (surgery and needle biopsy), 35 were malignant, and 9 were non-malignant. In terms of sensitivity, [^68^Ga]Ga-FAPI-RGD identified 32 lesions out of a total of 35, and [^18^F]FDG identified 27 (91.4% *vs.* 77.1%, *p* < 0.05, one-tailed test). [^68^Ga]Ga-FAPI-RGD also had higher accuracy than [^18^F]FDG (79.5% *vs.* 68.2%, *p* < 0.05). In addition, as compared to [^18^F]FDG, [^68^Ga]Ga-FAPI-RGD showed a higher tumor uptake (SUVmax, 6.9 ± 5.3 *vs.* 5.3 ± 5.4, *p* < 0.001) and higher T/B ratio (10.0 ± 8.4 *vs.* 9.0 ± 9.1, *p* < 0.05, respectively) in 42 malignant neoplasms (mean diameter 2.5 ± 1.9 cm) which was confirmed by pathology and 3-month follow-up, with a statistically significant difference.

### Comparison of [^18^F]FDG and [^68^Ga]Ga-FAPI-RGD in lung neoplasm metastases

**Figure 6:**
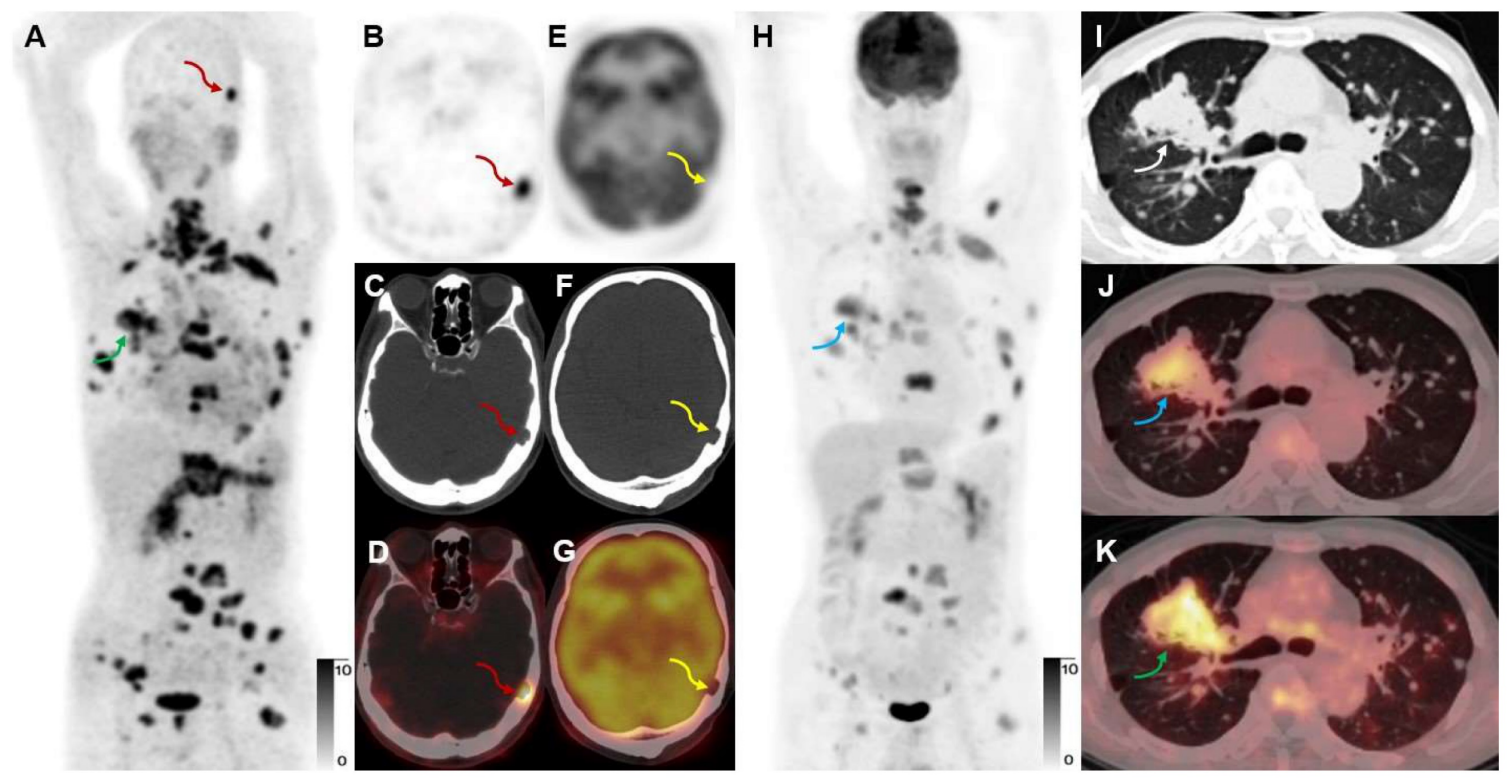
A 65-year-old male participant (Participant No. 9) was diagnosed with IAC with multiple lung, bone, and lymph node metastases at presentation. [^68^Ga]Ga-FAPI-RGD (A, K, green arrows) showed more lesions and a higher T/B ratio than [^18^F]FDG (H, J, blue arrows). Due to the low expression of FAP in the brain, a skull metastasis was clearly visualized on [^68^Ga]Ga-FAPI-RGD PET (A-D, red arrows, SUVmax 15.2), which showed no significant uptake on [^18^F]FDG PET (E-H, yellow arrows; the sections appeared different due to different head positions and the skull metastasis could be seen on CT alone which confirmed that these are comparable slices).

Eight untreated participants with advanced lung cancer received [^68^Ga]Ga-FAPI-RGD, 7 of whom had also undergone [^18^F]FDG PET/CT for a head-to-head comparison. [^68^Ga]Ga-FAPI-RGD and [^18^F]FDG were able to detect 254 and 220 neoplasms, respectively, in these 7 participants, which was not statistically significant (*p* > 0.05). We compared the SUVmax of the metastatic neoplasms detected by the two tracers. A paired analysis was performed for all positive lesions. As shown in Figure 6, [^68^Ga]Ga-FAPI-RGD PET/CT showed a higher SUVmax and T/B ratio for metastases than [^18^F]FDG, which was statistically significant (SUVmax, 12.2 ± 5.8 *vs.* 7.2 ± 3.5, *p* < 0.001, T/B ratio, 6.4 ± 3.0 *vs.* 3.8 ± 1.8, *p* < 0.001, respectively) (**Figure 6**).

### [^68^Ga]Ga-FAPI-RGD PET/CT and overexpression of FAP and integrin β_3_

**Figure 7:**
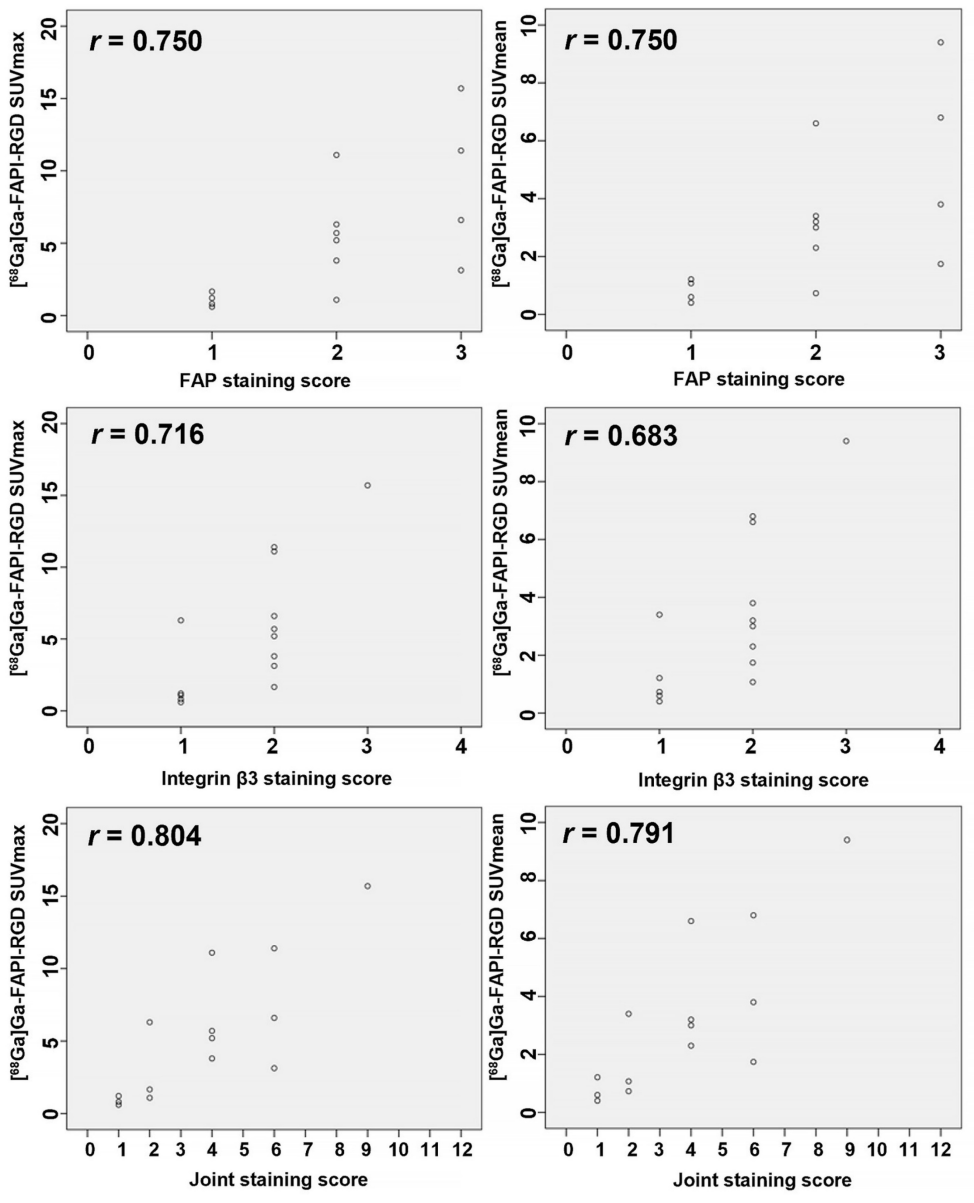
The Scatter plots and the Spearman correlation coefficient between FAP, integrin β_3_, and the FAP-integrin β_3_ joint staining score with SUVs of [^68^Ga]Ga-FAPI-RGD.

Overexpression of FAP and integrins α_v_β_3_ on tumor cells is the main factor by which [^68^Ga]Ga-FAPI-RGD shows high uptake in tumor and metastatic lesions. We evaluated the immunohistochemical staining of FAP and integrin β_3_ in the pathological sections of 14 primary lesions from 13 participants undergoing surgery. Expression of receptors *via* FAP or integrins β_3_ immunostaining intensity and percentage of positive tumor cells were scored. In this study, we also multiplied the staining score of FAP (0-3 score) and the staining score of integrin β_3_ (0-4 score) for the first time and named it “the Joint Score” (0-12 score). Our preliminary results showed that both SUVmax and SUVmean were positively correlated with the expression of FAP (*r* = 0.750 and 0.750, *p* < 0.01), integrins β_3_ (*r* = 0.716 and 0.683, p < 0.01), and the Joint Score (*r* = 0.804 and 0.791, *p* < 0.01), respectively (**Figure 7**).

### [^68^Ga]Ga-FAPI-RGD uptake compared with [^68^Ga]Ga-FAPI and [^68^Ga]Ga-RGD uptake in primary and metastatic lung neoplasms

**Figure 8:**
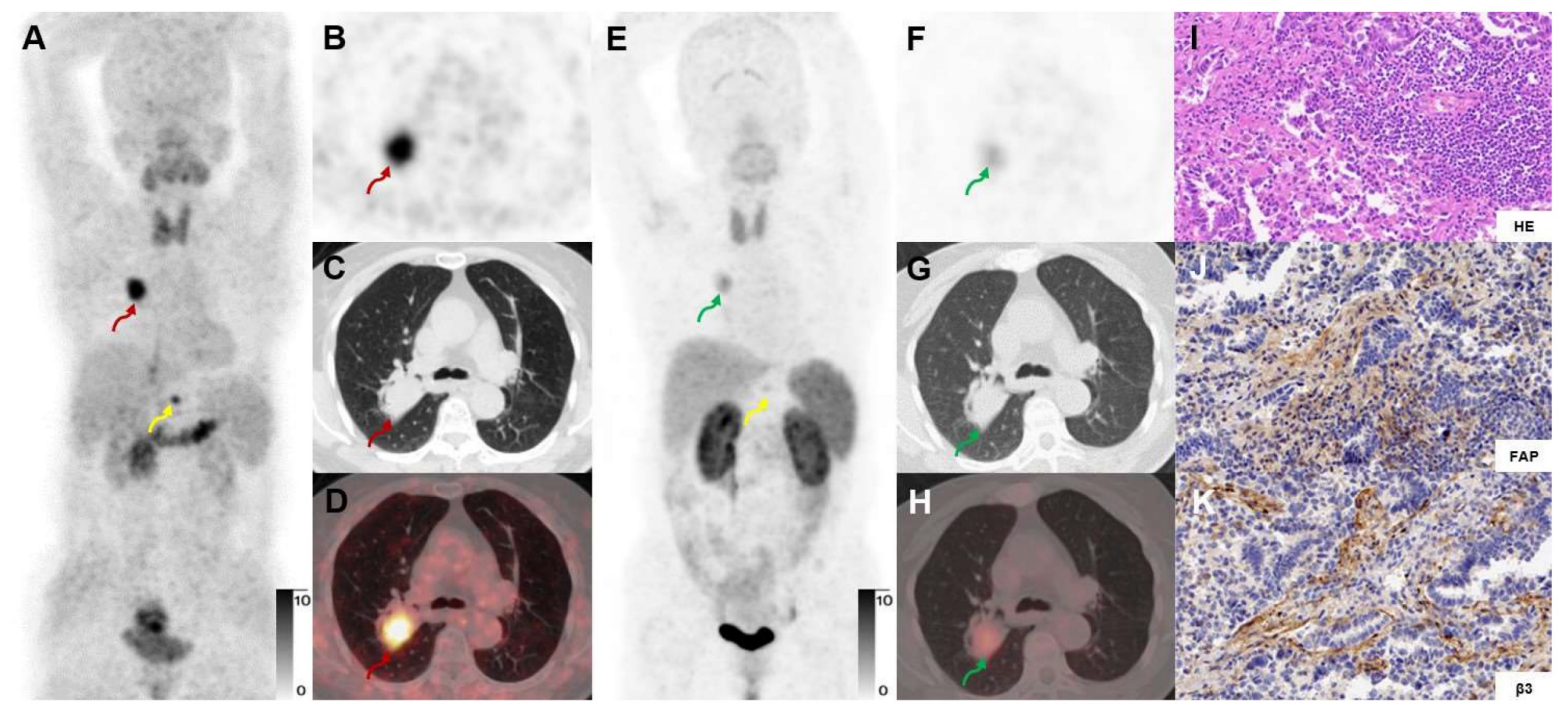
A 73-year-old woman with IAC (Participant No. 12) underwent two PET scans. The SUVmax of dual-target tracer [^68^Ga]Ga-FAPI-RGD (A-D) was visually higher than that of the single-target tracer [^68^Ga]Ga-RGD (E-H) in the right lung lesion (SUVmax, 11.1 *vs.* 3.4) and the T12 vertebral metastasis (yellow arrows). Immunohistochemical staining showed high expression of FAP and β_3_ (J, K).

**Figure 9:**
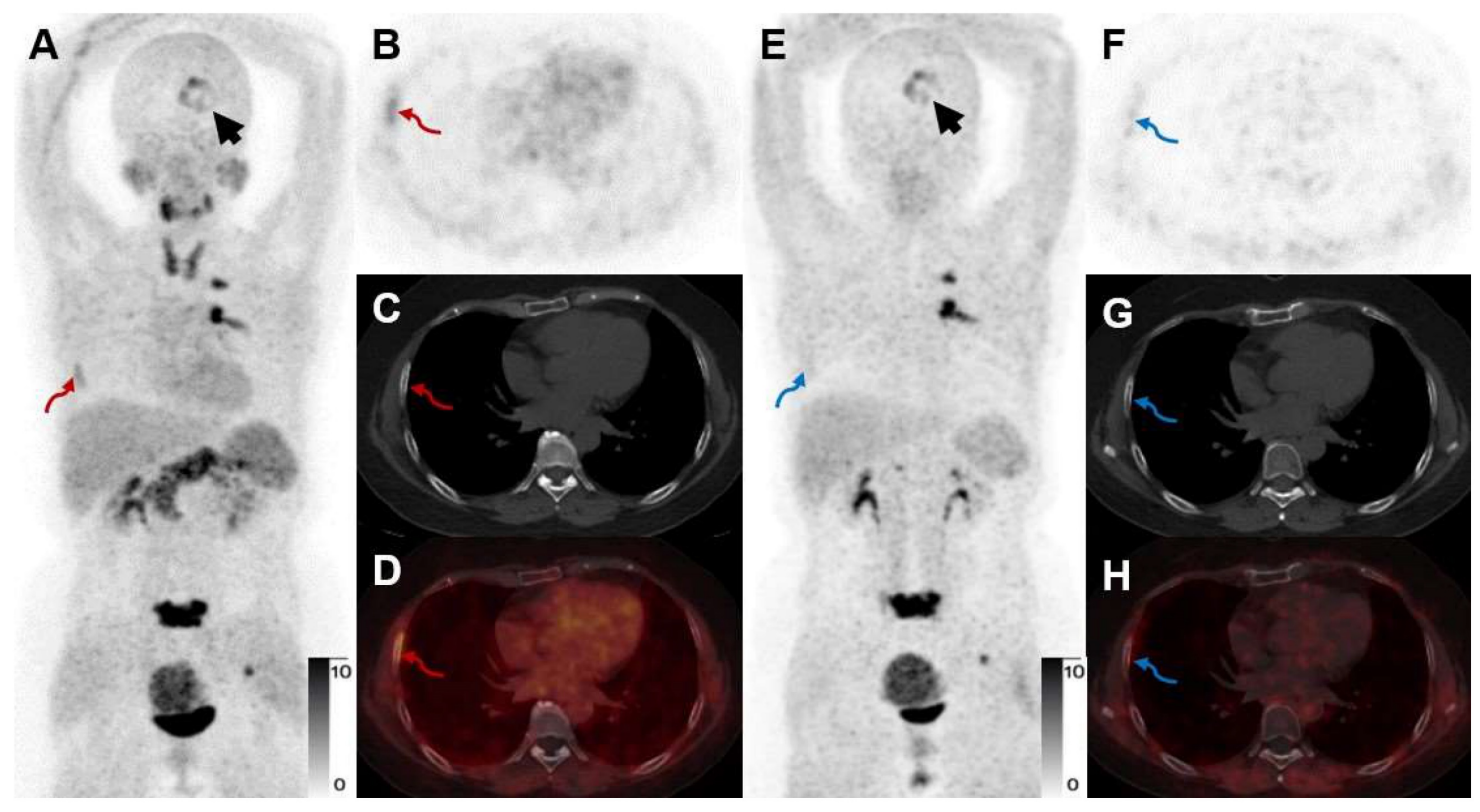
A 52-year-old woman with IAC (Participant No. 44) underwent two PET scans. [^68^Ga]Ga-FAPI-RGD showed higher uptake in brain metastasis than [^68^Ga]Ga-FAPI (SUVmax, 7.7 *vs.* 5.6, black arrows). One more rib lesion with high uptake was found (A-D, red arrows, SUVmax 4.5) by [^68^Ga]Ga-FAPI-RGD PET/CT while not detected by [^68^Ga]Ga-FAPI PET/CT (E-H, blue arrows), which had been confirmed as a metastatic lesion on previous whole-body bone scan.

For a head-to-head comparison with [^68^Ga]Ga-FAPI-RGD, 10 participants underwent [^68^Ga]Ga-RGD PET/CT. [^68^Ga]Ga-FAPI-RGD had a better detection rate of primary malignant tumors (sensitivity 100% *vs.* 80%, *p* < 0.05). Higher mean SUVmax and T/B ratio were observed for [^68^Ga]Ga-FAPI-RGD PET/CT scan compared with [^68^Ga]Ga-RGD PET/CT (SUVmax, 5.8 ± 4.4 *vs.* 2.3 ± 1.3, *p* < 0.001, T/B ratio, 6.1 ± 8.0 *vs.* 2.2 ± 5.0, *p* < 0.05, respectively). Data from one participant showed that the SUVmax of the dual-target tracer [^68^Ga]Ga-FAPI-RGD was higher than that of [^68^Ga]Ga-RGD in primary lung lesion (SUVmax, 11.7 *vs.* 3.7) and a metastatic bone lesion (**Figure 8**). Similarly, for comparison with [^68^Ga]Ga-FAPI PET/CT, we also had 9 participants who underwent both dual- and single-target tracer PET/CT scans. The SUVmax and T/B ratio of [^68^Ga]Ga-FAPI was slightly higher than that of [^68^Ga]Ga-FAPI-RGD in primary neoplasms (SUVmax, 7.2 ± 6.6 *vs.* 6.8 ± 5.6, T/B, 10.5 ± 9.5 *vs.* 10.3 ± 9.8, respectively) which was not statistically significant (*p* > 0.05). In a participant with both bone and brain metastases, however, [^68^Ga]Ga-FAPI-RGD had higher uptake (SUVmax, 7.7 *vs.* 5.6) in a brain metastasis than [^68^Ga]Ga-FAPI, and one more rib metastasis was found by [^68^Ga]Ga-FAPI-RGD PET/CT (SUVmax 4.5), which was [^68^Ga]Ga-FAPI negative (**Figure 9**).

## Discussion

[^18^F]FDG is now widely used in staging and restaging lung cancer 3-5. However, limitations, including low or no uptake in early malignant lesions and certain types of lung cancer, have been reported and need to be overcome in the light of potentially better tracers 10, 11. Several alternative tracers are therefore under development, with targets including gastrin-releasing peptide receptor, FAP, and integrin receptor α_v_β_3_ 20, 23, 34, 35. Past studies have already suggested the advantages of heterodimers over their constituent monomers; both preclinical and clinical studies showed that the binding affinity of the multivalent effect of the dimer structure is better due to more available receptors 24, 36, 37. Dual-targeting tracer [^68^Ga]Ga-FAPI-RGD had a significantly higher detection rate than [^18^F]FDG in detecting primary tumors, specificity in detecting mediastinal lymph nodes, and sensitivity in the detection of metastases. Compared with [^68^Ga]Ga-RGD PET/CT, [^68^Ga]Ga-FAPI-RGD had a better detection rate of primary tumors and a higher average SUVmax and T/B ratio. When compared with [^68^Ga]Ga-FAPI, the mean SUVmax of tumor lesions on [^68^Ga]Ga-FAPI-RGD PET/CT was not inferior to that on [^68^Ga]Ga-FAPI PET/CT.

We conducted this pilot exploratory study, based on the notable expression level of FAP and integrin α_v_β_3_ in lung cancer, in a cohort of participants with suspected lung cancer. Our study demonstrated that [^68^Ga]Ga-FAPI-RGD PET/CT, which traces tumor-specific receptors, may be used for identifying a more significant number of primary lung neoplasms and metastases. For micro-invasive adenocarcinomas and mucinous adenocarcinomas, we observed low uptake of both [^68^Ga]Ga-FAPI-RGD and [^18^F]FDG. The low uptake of [^18^F]FDG in these two types of tumors has earlier been described and might be associated with their histopathological characteristics 38, 39. To explain the low uptake of [^68^Ga]Ga-FAPI-RGD, the expression levels of FAP and integrin α_v_β_3_ need to be immunohistochemically investigated. Our study showed that in cases where [^18^F]FDG PET/CT performed little or no uptake of the lesions in several patients with early-stage lung cancer, these lesions were still positive with a mild to moderate uptake on [^68^Ga]Ga-FAPI-RGD PET scans, which would improve the value of PET/CT in the diagnosis of early-stage lung cancer (**Figure 4-5**).

In our previous studies on this tracer, we showed that by targeting both FAP and integrin α_v_β_3_, the radiotracer [^68^Ga]Ga-FAPI-RGD had excellent tumor-specific uptake, prolonged tumor retention time, as well as favorable targeting ability and pharmacodynamics in preclinical xenograft models; in our first-in-human biodistribution study we preliminarily indicated that the new tracer has a promising diagnostic capability compared with [^18^F]FDG regarding T/B ratio and sensitivity, we observed rapid and high tumor uptake, long tumor retention and high tumor to background ratio in the 0-3h p.i. biodistribution 26, 31. Furthermore, we found that the lesion uptake continued to increase at 2-3h **(Table S1 and Figure 3)**. In contrast, the normal organ uptake decreased, suggesting that the new tracer has long tumor retention, making it suitable for potential cancer therapy if labeled with a therapeutic isotope. There are already many studies proving the feasibility of using ^177^Lu labeled FAPI tracers as cancer treatments, although the low tumor retention remains an issue to be solved; our study provided another tracer design strategy with great potential to improve ^177^Lu labeled FAPI radioligand therapy 40.

In the head-to-head comparison with [^68^Ga]Ga-RGD in 10 participants, primary tumor uptake was significantly higher for [^68^Ga]Ga-FAPI-RGD. This was not observed when comparing [^68^Ga]Ga-FAPI-RGD with [^68^Ga]Ga-FAPI in the other 9 participants, where [^68^Ga]Ga-FAPI-RGD was found to be non-inferior. We speculated that this could be due to the relatively larger molecular size of [^68^Ga]Ga-FAPI-RGD; a study on TATE-RGD by Liu *et al.* found similar results 41. A major drawback of [^68^Ga]Ga-FAPI-RGD was the high physiological uptake to the thyroid gland and pancreas, similar to other reported FAPI dimer compounds 26, 42. However, thyroid uptake is not receptor-specific, and the mechanism of this unspecific uptake is yet to be established.

Previous studies have shown that lung cancer can express both FAP and integrin receptors, which was also confirmed in our study 16, 17, 28, 43, 44. Different staining levels for both receptors were observed in all lung cancer sections. Although we found that FAP, integrin β_3_ staining scores, and the FAP-integrin β_3_ Joint Score were all positively correlated with SUVs of [^68^Ga]Ga-FAPI-RGD, we must acknowledge that the small sample size of our pathological section cohort did not allow us to draw a strong conclusion and that results need to be confirmed in future studies by histopathological analysis of larger lung cancer tissue samples. No previous studies have stained for both FAP and integrin receptors and investigated the differences in their expression and correlation of expression with different stages of lung cancer. A dual-targeting tracer in PET imaging can potentially overcome this challenge of a variable receptor expression. In this study, we presented an innovative staining score and found that it had a slightly higher correlation coefficient with SUVs than did FAP and integrin β_3_ individually, although both the percentage of positively stained cells (PP) and the staining intensity (SI) correlate with SUV values. In the future, more pathological tissue samples will be needed to investigate the relationship between IHC staining and the uptake values of [^68^Ga]Ga-FAPI-RGD.

The limited number of enrolled participants may have been insufficient to fully demonstrate this new PET tracer's diagnostic value. For ethical reasons, the same participant could not receive both the dual-targeting tracer and the two single-targeting tracers for comparison, and a larger cohort is needed in future studies for these head-to-head comparisons to reach a conclusive significant difference. Patients with different stages of lung tumors may present differently on [^68^Ga]Ga-FAPI-RGD PET/CT and other PET tracers evaluated, requiring more patients to be included for subgroup analysis and comparison. Only puncture biopsy samples were acquired for some participants with advanced lung cancer. As there was a lack of tissue samples to perform complete immunohistochemical validation, diagnosis of lymph nodes and distant metastases was merely based on imaging. In addition, the participants in this cohort were predominantly diagnosed with adenocarcinomas; a study comprehending a more heterogeneous cohort of lung cancer participants needs to be done.

## Conclusion

Our proof-of-concept study supports using PET/CT with the dual-target tracer [^68^Ga]Ga-FAPI-RGD in diagnosing and staging lung cancer. We found that [^68^Ga]Ga-FAPI-RGD may have better sensitivity in detecting lung cancer than the other PET tracers evaluated. We thus conclude that [^68^Ga]Ga-FAPI-RGD is a feasible tracer worth further investigation; the data suggest that [^68^Ga]Ga-FAPI-RGD PET/CT may be a promising approach to detect lung cancer better. We acknowledge that more comprehensive studies are needed in order to reach a robust conclusion. In addition, due to the rapid clearance and inadequate tumor retention of monomeric FAPIs, FAPI-RGD should be explored for potential therapeutic use in future studies.

## Supplementary Material

Supplementary table 1: the SUVmean values for lesions and normal organs.

## Figures and Tables

**Table 1:** Participant Demographics and Clinical Characteristics

No.	Gender	Age (years)	Smoking history	LD (cm)	Pathologic diagnosis		Follow-up	[^68^Ga]Ga-FAPI-RGD	[^18^F]-FDG	[^68^Ga]Ga-FAPI	[^68^Ga]Ga-RGD
					Surgery	Puncture					
1	M	70	Yes	4.4		IAC		P	P		
2	M	72	Yes	6		IAC		P	P		
3	M	43	No	3.2	IAC			P	P		
4	F	46	No	0.7	MIA			P	N		N
			No	2	Hamartoma			P	P		N
5	F	39	No	0.7	AAH			N	N		
6	F	30	No	0.8	IAC			P	P		
7	M	66	No	1.1	AIS			N	N		
				0.8	AIS			N	N		
8	F	63	No	1.7	IAC			P	P		P
				1.0	MIA			N	N		N
9	M	65	Yes	6.2		IAC		P	P		
10	M	79	Yes	10		PSC		P	P		
11	F	69	No	2.5	IAC			P	P		
12	F	72	No	3.8	IAC			P	P		P
13	F	71	No	1.6	MIA			N	P		P
				1	AAH			N	N		N
14	M	66	Yes	3		IAC		P	P		
15	F	66	No	3.2	IAC			P	P		P
16	F	41	Yes	1.7	IAC			P	P		
17	F	79	No	2.6	IAC			P	P		P
				1.7	IAC			P	P		P
18	M	67	Yes	1.7	IAC			P	P		
19	F	50	No	0.6	IAC			P	P		
20	F	66	No	3.1		IAC		P	P		
21	F	54	No	2.7		IAC		P	P		
22	F	48	No	2.9	IAC			P	P		
23	M	57	Yes	7.7		SC		P	P	P	
24	M	51	Yes	1.1	IAC			P	P		
25	F	68	No	0.7	MIA			P	N		
				0.8	AIS			P	N		
							CT	P	N		
26	F	70	No	2.1	IAC			P	P	P	
				1.4	AIS			P	N	P	
27	F	67	No	1.6		IAC		P	P		
28	F	75	No	2.2		IAC		P	P		
29	F	70	No	1.3	IAC			P	P	P	
30	F	47	No	3.2		SC		P	P		
31	F	34	No	1.2	IAC			P	P	P	
32	F	79	No	1.7			CT	P	P		
33	M	78	Yes	2.2			CT	P	P		
34	M	57	Yes	2.0			CT	P	P	P	
35	F	58	No	2.4			CT	P	P		
36	M	69	Yes	2.5			CT	P	P		
37	M	56	Yes	0.4	Inflammation			N	N		P
38	M	73	Yes	1.6	Inflammation			P	P	P	
				0.9	Inflammation			P	P	P	
39	F	41	No	1.8	Inflammation			P	P		
40	F	69	No	1.3	PL			P	P		
41	F	36	No	1.3		Inflammation		P	P		
42	M	74	No	4.5	IAC			P			P
43	M	61	Yes	2			CT	P			N
44	F	52	No	1.9		IAC		P		P	
45	F	60	Yes	2.2		IAC		P		P	
46	M	67	Yes	1.9			CT	P		P	
47	M	58	Yes	2.8		SPT		P	P		
48	M	71	No	1.6			Lost	N	N		
49	M	56	Yes	1.5			Lost	P	P		
50	F	64	No	3.8		IAC		P			
51	F	69	No	4.2		IAC		P			

AAH: atypical adenomatous hyperplasia; AIS: adenocarcinoma *in situ*; CT: follow-up by chest CT; Hamartoma: pulmonary hamartoma; Inflammation: pulmonary infection; LD: length diameter; Lost: lost to follow-up; IAC: invasive adenocarcinoma cancer; MIA: minimally invasive adenocarcinoma; N: negative; P: positive; PSC: pulmonary sarcomatoid carcinoma; PL: pulmonary leiomyoma; SC: squamous cell lung cancer; SPT: second primary tumor.

**Table 2:** Estimated organ-absorbed doses of [^68^Ga]Ga-FAPI-RGD (mSv/MBq)

Target Organ	Mean	SD
Adrenals	1.60E-02	2.23E-03
Brain	9.60E-03	1.10E-03
breasts	9.94E-03	9.79E-04
Esophagus	1.15E-02	1.05E-03
Eyes	9.56E-03	1.12E-03
Gallbladder Wall	1.25E-02	8.07E-04
Left colon	1.24E-02	1.23E-03
Small Intestine	1.21E-02	1.12E-03
Stomach Wall	1.26E-02	1.17E-03
Right colon	1.18E-02	1.13E-03
Rectum	1.39E-02	2.24E-03
Heart Wall	3.21E-02	7.16E-03
Kidneys	3.42E-02	7.82E-03
Liver	2.03E-02	8.53E-03
Lungs	1.10E-02	1.13E-03
Ovaries	1.34E-02	1.46E-03
Pancreas	7.29E-02	2.49E-02
Prostate	6.56E-02	2.88E-02
Salivary Glands	4.91E-02	1.59E-02
Red Marrow	2.06E-02	3.92E-03
Osteogenic Cells	1.43E-02	3.19E-03
Spleen	3.42E-02	1.00E-02
Testes	9.58E-03	7.16E-04
Thymus	3.48E-02	4.20E-02
Thyroid	7.80E-02	5.13E-02
Urinary Bladder Wall	8.00E-02	3.25E-02
Uterus	4.71E-02	2.32E-02
Total Body	1.22E-02	1.10E-03
Effective Dose	2.02E-02	1.80E-03

## Abbreviations

FAPI: fibroblast activation protein inhibitor
RGD: Arg-Gly-Asp
FDG: fluorodeoxyglucose
SUVmax: maximum standardized uptake value
SUVmean: mean standardized uptake value
