# Supplementary material for: Dual targeting PET tracer [68Ga]Ga-FAPI-RGD in patients with lung neoplasms: a pilot exploratory study
Source: Theranostics. 2023 May 15;13(9):2979–92. doi: 10.7150/thno.86007 (PMC10240811; doi:10.7150/thno.86007)
Supplement: Supplementary file 1 — Supplementary table 1: the SUVmean values for lesions and normal organs. [file thnov13p2979s1.pdf]

		lesion	mediastinum	liver	muscle	pancreas	thyroid	parotid gland	salivary gland	uterus	kidney	spleen	red marrow	lung
patient1	5-15min	4.8	8.2	5.3	1.9	13.5	13.5	5.9	7.3	5.4	5.81	5.92	3.13	1.35
	20-30min	4.8	7.1	4.8	1.7	11.8	11.8	5	7.6	4.3	5.47	5.7	2.82	1.11
	35-45min	4.4	6.7	4.1	1.9	10	10	4.9	7.1	6	5.21	5.16	2.75	0.87
	50-60min	4.7	5.4	4.1	1.8	9.1	9.1	4.9	6.8	5.8	4.93	5.11	2.7	0.98
	70-90min	4.5	4.5	3.6	1.9	7.9	7.9	4.5	7.4	7.5	4.35	4.25	2.78	1
	100-120min	5.7	3.6	3	1.6	6	6	3.6	6.3	7.9	3.25	3.59	1.92	0.73
	130-150min	5.5	2.6	3.2	2.1	5.6	5.6	3.1	5.7	9.3	3.52	3.54	1.95	0.52
	160-180min	5.2	1.8	3.4	2	4.2	4.2	2.9	4.4	10.1	3.37	3.4	1.66	0.53
		**lesion**	**mediastinum**	**liver**	**muscle**	**pancreas**	**thyroid**	**parotid gland**	**salivary gland**	**prostate**	**kidney**	**spleen**	**red marrow**	**lung**
patient2	5-15min	6.7	4.4	2.2	0.8	10.7	9.4	3.5	6.4	3.23	5	3.73	2.31	0.67
	20-30min	7.1	4.1	2.2	1	10.1	8.9	2.9	6.2	3.27	3.88	3.5	2.27	0.62
	35-45min	7.1	3.7	2.1	1	10	8.9	3	6.3	3.04	3.51	3.36	2.09	0.54
	50-60min	6.9	3.2	2	1	9.2	8.1	3	6.4	2.4	4.34	3.13	2.18	0.58
	70-90min	8.5	2.3	1.7	1.1	9.2	7.4	3.2	6	2.33	3.17	2.95	1.78	0.52
	100-120min	8.7	1.8	1.5	1	7.7	6	2.6	5.7	1.93	2.83	2.74	1.77	0.53
	130-150min	8.7	1.6	1.7	1.4	6.5	4.9	3	5.1	1.74	2.8	2.33	1.31	0.51
	160-180min	8.9	1.6	1.6	1.4	5.2	4.6	2.9	5	1.77	2.24	2.47	1.32	0.43
		**lesion**	**mediastinum**	**liver**	**muscle**	**pancreas**	**thyroid**	**parotid gland**	**salivary gland**	**prostate**	**kidney**	**spleen**	**red marrow**	**lung**
patient3	5-15min	5.3	5.5	3.1	1.1	9.1	12.8	3	6.5	3.35	5.96	3.77	2.54	1
	20-30min	5.5	5.1	2.5	1	8.6	9	3.2	5.9	3.15	4	3.53	2.56	0.83
	35-45min	5.7	4.4	2.6	1.1	7.9	10	3	5.8	4.33	3.53	3.3	2.64	0.71
	50-60min	6.2	3.6	2.2	1.2	7	8.2	3.1	6.3	2.84	4.48	3.83	2.37	0.78
	70-90min	7.2	2.9	1.8	1.2	5.3	6.5	2.9	5.8	2.22	2.87	3.39	1.78	0.56
	100-120min	6.8	2.3	1.4	1.4	4.4	5	2.9	5.1	2.32	2.57	2.87	1.52	0.35
	130-150min	7.6	1.6	1.4	1.5	3.3	3.7	2.6	4.5	1.39	2.39	2.11	1.47	0.33
	160-180min	8	1.2	1.1	1.5	3	3.6	2.3	4.3	1.58	2.81	2.3	1.46	0.37
		**lesion**	**mediastinum**	**liver**	**muscle**	**pancreas**	**thyroid**	**parotid gland**	**salivary gland**	**prostate**	**kidney**	**spleen**	**red marrow**	**lung**
patient4	5-15min	3.7	5.6	3.6	0.9	8.1	13.7	3.3	6.8	2.9	3.82	4.04	2.16	1.57
	20-30min	3.8	5.5	3.2	0.9	8.2	14.6	3.1	6.8	2.46	3.85	3.63	2.38	1.27
	35-45min	3.8	4.8	3	1.1	8.7	11.8	3.4	6.9	2.64	3.47	3.23	1.94	1.16
	50-60min	4.8	4.1	2.8	1	7.8	13.9	3.2	7.1	2.28	3.76	2.83	2.06	1.36
	70-90min	5.3	3.2	2.3	0.9	6	11.9	3.6	6.6	2.65	3.73	2.63	1.66	1.31

	100-120min	4.7	2.3	2.5	1.2	5.8	7.9	3.7	6.6	2.57	3.82	2.57	1.49	0.91
	130-150min	5.8	1.9	1.8	1.2	4.7	8.7	3.1	6.3	2.18	3.17	2.1	11.41	0.65
	160-180min	5.5	1.9	1.9	1.4	5.6	6.5	2.8	5.7	2.37	3.6	2.5	1.27	0.53
		**lesion**	**mediastinum**	**liver**	**muscle**	**pancreas**	**thyroid**	**parotid gland**	**salivary gland**	**prostate**	**kidney**	**spleen**	**red marrow**	**lung**
	5-15min	7.8	6.1	2.8	0.9	7	5.9	3.3	4.7	2.72	5.59	4.16	2.21	0.66
	20-30min	9.3	5.5	2.5	0.7	6.4	5.5	2.6	4.5	2.26	4.67	4.05	2.2	0.76
	35-45min	9.1	4.8	2.3	0.6	6.1	4.7	2.5	4.2	2.51	4.56	3.49	2.03	0.83
**patient5**	50-60min	9.9	4	2.2	0.7	5.6	5.4	2.4	3.7	2.51	4.55	3.41	2.33	0.41
	70-90min	10	3	2.1	0.8	6.3	5.2	2.8	5.1	2.5	4.13	3.4	2.02	0.52
	100-120min	11.1	2.5	1.8	0.9	4.7	5	2.7	4.9	2	3.87	2.92	1.6	0.39
	130-150min	13.2	1.8	1.5	0.9	5	4.6	2	4.2	1.99	3.78	2.47	1.27	0.57
	160-180min	14.3	1.6	1.4	0.9	4.3	4.7	2.1	5.5	1.76	3.16	2.31	1.42	0.34
		**lesion**	**mediastinum**	**liver**	**muscle**	**pancreas**	**thyroid**	**parotid gland**	**salivary gland**	**uterus**	**kidney**	**spleen**	**red marrow**	**lung**
	5-15min	7.9	6.1	4.8	1.2	9.7	10.9	2.3	4.6	-	8.58	4.66	2.33	1.41
	20-30min	7.9	5.3	4.6	1.1	8.4	9.8	2.2	4.2	-	4.92	4.06	1.97	1.24
	35-45min	8.6	4.4	4.3	1.2	8.2	8.8	2.3	4.4	-	4.85	3.74	1.9	1.07
**patient6**	50-60min	9	4	3.6	1.4	8.3	8.4	2.5	4.7	-	3.52	3.33	1.56	0.77
	70-90min	11.1	3	3.8	1.6	6.9	7.3	2.5	4.7	-	3.43	3.42	1.69	0.65
	100-120min	11.1	2.3	3.9	1.7	6.1	5.7	2.3	4.7	-	3.41	2.87	1.24	0.57
	130-150min	13.8	2	4	2.1	6	5	2.8	5.3	-	-	-	-	-
	160-180min	13	1.6	3.2	1.6	4.2	3.7	2.5	4.7	-	2.78	2.96	1.25	0.32
		**lesion**	**mediastinum**	**liver**	**muscle**	**pancreas**	**thyroid**	**parotid gland**	**salivary gland**	**uterus**	**kidney**	**spleen**	**red marrow**	**lung**
	5-15min	3.2	6.7	2.5	1.1	10.2	10.7	2	5.9	2.1	8.62	4.2	1.54	1.17
	20-30min	2.8	6	2	1.1	9.2	10.1	1.9	5.5	2.5	5.1	3.88	1.42	0.98
	35-45min	3.7	5.1	2	1.1	9.3	9.2	2.1	5.3	2.1	4.56	3.42	1.46	0.86
**patient7**	50-60min	3.5	4.8	2.2	1.1	8.5	9.8	2.2	5.4	2.2	4.13	3.56	1.55	0.79
	70-90min	3.7	3.7	1.8	1.1	7.2	7.8	2.1	5.7	2.4	3.91	3.06	1.17	0.76
	100-120min	3.8	3	1.4	1.4	6	6.4	2.2	5.3	2.8	3.48	2.61	1.24	0.71
	130-150min	5.3	2.1	1	1.4	5.4	6.1	2.2	6.5	3.1	5.99	2.33	1.38	0.56
	160-180min	4.6	2.2	0.8	1.6	5.3	4.4	2	5.3	2.9	4.62	2.28	1.28	0.52
		**lesion**	**mediastinum**	**liver**	**muscle**	**pancreas**	**thyroid**	**parotid gland**	**salivary gland**	**uterus**	**kidney**	**spleen**	**red marrow**	**lung**
	5-15min	2.9	4.4	2.4	1	7.2	12.3	4.6	8.7	6.5	8.51	3.09	2.34	1.45
	20-30min	2	4.1	2.5	1	6.8	11.7	4.4	8.4	7.1	3.85	2.78	2.21	1.12

		lesion	mediastinum	liver	muscle	pancreas	thyroid	parotid gland	salivary gland	uterus	kidney	spleen	red marrow	lung
patient8	35-45min	2.3	3.5	2.5	1	6.1	10.5	4.5	8.2	8.4	4.13	2.74	2.39	1.13
	50-60min	2.3	2.9	2.1	1.2	6.1	8.8	4.2	7.5	7.2	3.27	2.69	2.39	1
	70-90min	3.3	2.3	1.3	1.1	4.9	7.8	4.3	7.3	8.4	2.92	2.03	1.89	0.98
	100-120min	3.4	1.9	1.1	1.2	4	5.9	3.9	6.6	8.4	2.42	1.88	1.92	0.91
	130-150min	3.8	1.6	1	1.3	3.2	5.3	3.5	6.7	8.3	3.09	1.77	1.24	0.32
	160-180min	-	-	-	-	-	-	-	-	-	-	-	-	-

		lesion	mediastinum	liver	muscle	pancreas	thyroid	parotid gland	salivary gland	uterus	kidney	spleen	red marrow	lung
patient9	5-15min	8.6	4.9	2.6	1.2	13.1	12.7	2.5	7.7	6.4	37.83	3.46	2.59	0.72
	20-30min	9	4	2.2	1.4	13.6	10	2.7	8	6.5	17.69	3.07	2.28	0.78
	35-45min	9.5	3.7	2.7	1.4	11.7	10.3	2.3	7.5	6.5	7.7	2.86	2.14	0.8
	50-60min	9.3	3.2	2.2	1.5	10.7	8.6	2.6	7.6	7.2	7.61	2.69	1.97	0.7
	70-90min	9.8	2.5	1.7	1.5	7.4	8	2.8	7.5	6.6	5.35	2.36	1.71	0.64
	100-120min	9.3	1.6	1.5	1.4	6.5	4.9	2.3	6.3	7.3	4.05	2.18	1.18	0.53
	130-150min	9.8	1.5	1.6	1.7	5	4.7	2.6	6.1	7.4	4.32	2.52	1.01	0.63
	160-180min	10.4	0.9	1.3	1.6	3.1	3.2	2.1	5.2	7.6	4.17	2.41	0.64	0.55
